# On Superstitions Connected with the History and Practice of Medicine and Surgery

**Published:** 1844-07

**Authors:** 


ART. IV.—*On Superstitions connected with the History and Practice of Medicine and Surgery.* By T. J. PETTIGREW, F.R.S.—*London*, 1844. 8vo, pp. 198.

THIS work is a literary one, belonging to the "belles lettres," the "polite learning" of medicine. It contains the result of various and extensive reading on alchymy, astrology, talismans, amulets, charms, on the royal touch, and other similar superstitions, by which the body has been influenced, through the medium of the mind. It is the common-place book of a literary physician with a running commentary, giving a unity and continuity to the mosaic. And although it has no directly practical aim, yet it is one of a class of books which we trust will not, in this practical age, be wholly superseded: for on such studies, in due subordination to more important ones, lead not alone to wider and less contracted views, (for "ignorance of all antiquity is a mutilation of the human mind,") but are essential to every profession which is considered a learned one. And the reputation for learning in our calling must mainly depend on the few whose tastes, industry, and early good education enable them to overcome the inducements to literary indolence, which the practice of medicine presents. On this account we hail such books as Mr. Pettigrew's. Whilst there is so much conversation on the position which medicine holds in this country, we cannot sufficiently remember that this depends mainly on each individual himself. No parliamentary enactments can raise into public estimation a body of unworthy men, or depress a class of superior men; and, unless each individual of our body assiduously cultivates his mind, and keeps himself at least on the level of the best educated classes, he himself must sink into a tradesman, and help to drag down the whole body. The habits of reading, which we get when students, should never be given up; it is one of those habits kept up, when once acquired, without much difficulty; but if not, not easily, if ever, regained. And unless a man, especially in the country, continually sharpens his own mind by collision with the minds of others of a higher grade than his own, and he can often only do that by means of books, he will gradually deteriorate.

Now we are on this subject, we cannot forbear quoting the following reminiscences from the writings of a man who, although actively employed in a laborious profession, was a most ardent student throughout his long life—the late Mr. Charles Butler. He says of himself:—

"Very early rising,—a systematic division of his time,—abstinence from all company, and from all diversions not likely to amuse him highly,—from reading, writing, or even thinking on modern party politics,—and, above all, never permitting a bit or scrap of time to be unemployed,—have supplied him with an abundance of literary hours. His literary acquirements are principally owing to the rigid observance of four rules: to direct his attention to one literary object only at a time; to read the best book upon it; consulting others as little as possible,—where the subject was contentious, to read the best book on each side; to find out men of information, and when in their society, to listen, not to talk."

And he adds,

"It is pleasing to him to reflect, that, though few have exceeded him in the love of literature, or pursued it with greater delight, it never seduced, or was suspected by his professional friends of seducing him, for one moment, from professional duty."

